# First person – Kalyanasundaram Parthasarathy

**DOI:** 10.1242/bio.058685

**Published:** 2021-03-26

## Abstract

First Person is a series of interviews with the first authors of a selection of papers published in Biology Open, helping early-career researchers promote themselves alongside their papers. Kalyanasundaram Parthasarathy is first author on ‘Spatial odor discrimination in the hawkmoth, *Manduca sexta* (L.)’, published in BiO. He conducted the research described in this article while a Senior Research Associate in Professor Mark A Willis's lab at the Department of Biology, Case Western Reserve University, Cleveland, Ohio, USA. He is now a Postdoc in the lab of Professor Sanjay P Sane at National Centre for Biological Sciences at the Tata Institute of Fundamental Research, Bangalore, India, investigating how animals use sensory cues to navigate, the related behaviours and the underlying brain function.


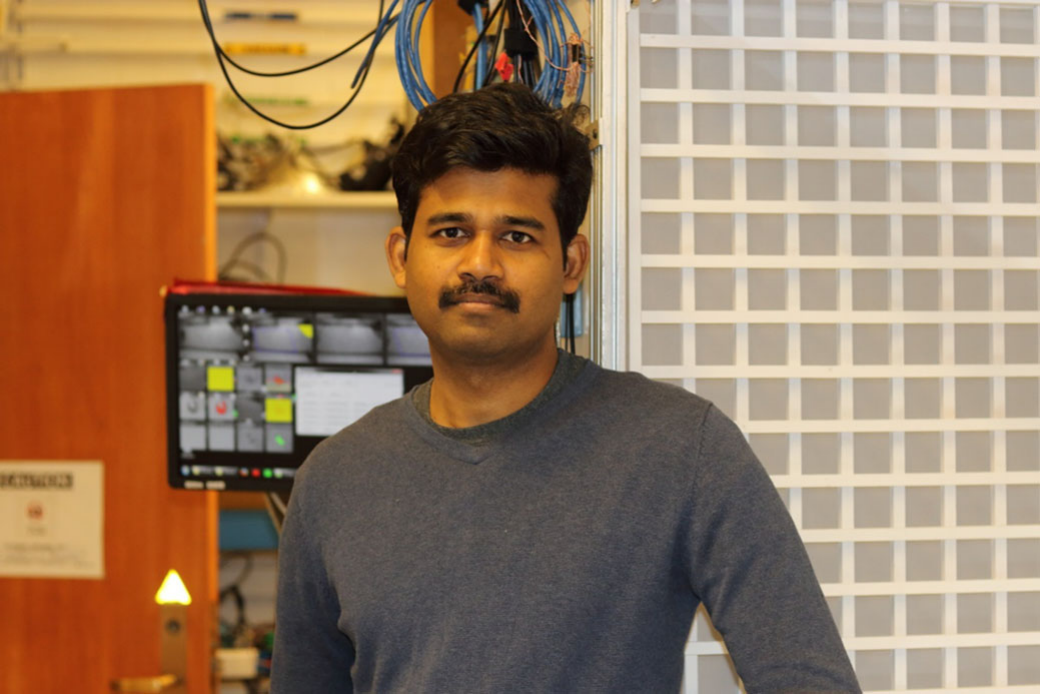


**Kalyanasundaram Parthasarathy**

**What is your scientific background and the general focus of your lab?**

I did my PhD thesis research in the field of mammalian olfaction and was keen to explore how animals can track odors in three dimensions and somewhat turbulent conditions, which is an exponentially more difficult task. To study this, I joined as a postdoctoral fellow in the laboratory of Prof. Mark Willis at the Case Western Reserve University, where I studied odor-driven navigation in nocturnal insects, which are superb at tracking very low concentration odor plumes in three dimensions in windy conditions. More recently, as a visiting fellow in Prof. Sanjay Sane's laboratory at the National Centre for Biological Sciences, I have been studying how insects combine odor plume tracking with visual recognition to track conspecific mates.

**How would you explain the main findings of your paper to non-scientific family and friends?**

In this study, we tested whether moths can tell the difference between smelling an odor on their left versus right antenna. To answer this question, I developed a learning test based on one originally designed to study odor learning in honeybees. The moths were given sugar water reward every time we puffed a flower odor on a single antenna i.e., either left or right side. After several repeats of this ‘pairing’ of flower odor on to a specific antenna and sugar reward, moths started to try to feed because they had learned to expect the sugar reward upon smelling the odor on the paired antenna. After they showed they had learned this pairing, we challenged moths by presenting the same flower odor to the ‘unpaired’ antenna i.e., not conditioned with the sugar water reward. We expected the moths to generate a feeding response when we applied the flower odor to the paired antenna side and not to generate feeding response for the unpaired antenna. As expected, when tested with the odor on the unpaired antenna moths showed no feeding response. The results from this work show that moths can discriminate the odor arrival side between the paired and unpaired antenna and thus can extract spatial odor information.

“… we tested whether moths can tell the difference between smelling an odor on their left versus right antenna.”

**What are the potential implications of these results for your field of research?**

Though the odor plume tracking behavior is a well-studied topic in flying moths and flies tracking attractive odors, the role of bilateral odor sampling during plume tracking flight is not fully understood. Our study is a first step in addressing the contribution of bilateral sampling in extracting spatial odor information in moths.

**Figure:**
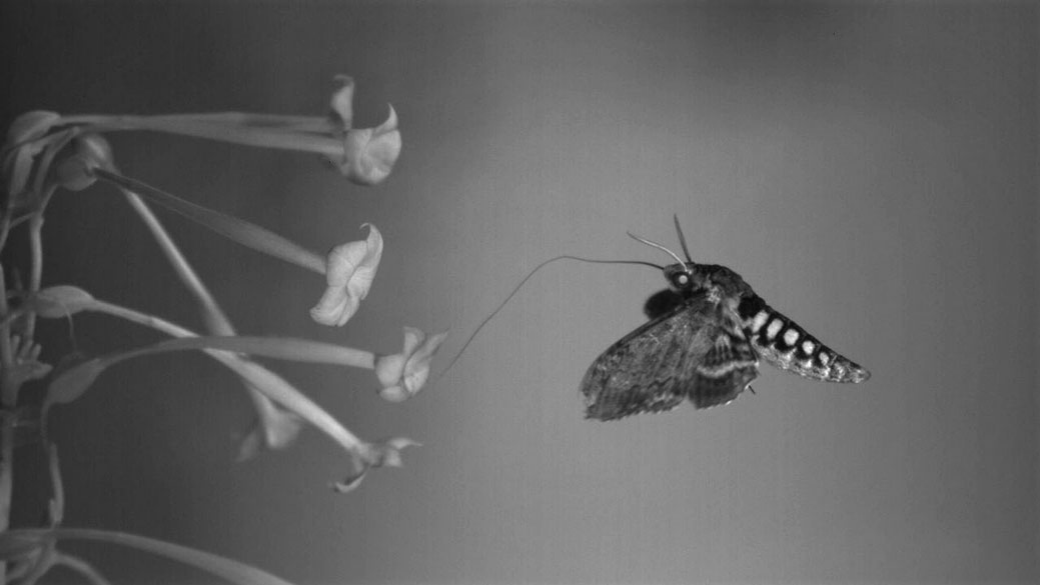
**A hawkmoth, (*Manduca sexta*) feeding on a tobacco flower.**

**What's next for you?**

The current study demonstrated that moths have the ability to recognize odor arrival direction. Our next set of experiments are aimed at studying the ability of moths to discriminate odor stimulus based on inter-antennal odor arrival time difference and concentration difference to further understand the role of spatial odor information in plume tracking. Throughout my research career, I have specifically focused on how chemical signals in the form of odor trails or pheromone plumes guide animals towards food or mates. I would like to pursue my research interest as an independent scientist in India.

“… moths have the ability to recognize odor arrival direction …”
